# Ideal Cardiovascular Health in Young Adult Populations From the United States, Finland, and Australia and Its Association With cIMT: The International Childhood Cardiovascular Cohort Consortium

**DOI:** 10.1161/JAHA.113.000244

**Published:** 2013-06-21

**Authors:** Mervi Oikonen, Tomi T. Laitinen, Costan G. Magnussen, Julia Steinberger, Alan R. Sinaiko, Terence Dwyer, Alison Venn, Kylie J. Smith, Nina Hutri‐Kähönen, Katja Pahkala, Vera Mikkilä, Ronald Prineas, Jorma S. A. Viikari, John A. Morrison, Jessica G. Woo, Wei Chen, Theresa Nicklas, Sathanur R. Srinivasan, Gerald Berenson, Markus Juonala, Olli T. Raitakari

**Affiliations:** 1 Research Centre of Applied and Preventive Cardiovascular Medicine, University of Turku, Finland (M.O., T.T.L., C.G.M., K.P., M.J., O.T.R.); 2 Menzies Research Institute Tasmania, University of Tasmania, Hobart, Tasmania, Australia (C.G.M., A.V., K.J.S.); 3 Department of Pediatrics, University of Minnesota Amplatz Children's Hospital, Minneapolis, MN (J.S., A.R.S.); 4 Environmental and Genetic Epidemiology Research Group, Murdoch Children's Research Institute, Royal Children's Hospital, Melbourne, Victoria, Australia (T.D.); 5 Department of Pediatrics, University of Tampere and Tampere University Hospital, Tampere, Finland (N.H., M.J.); 6 Paavo Nurmi Centre, Sports and Exercise Medicine Unit, Department of Physical Activity and Health, Turku, Finland (K.P.); 7 Department of Food and Environmental Sciences, University of Helsinki, Finland (V.M.); 8 Division of Public Health Sciences, Wake Forest University School of Medicine, Winston‐Salem, NC (R.P.); 9 Department of Medicine, University of Turku and Turku University Hospital, Finland (J.A.V.); 10 The Heart Institute, Department of Pediatrics, Cincinnati Children's Hospital Medical Center, Cincinnati, OH (J.A.M., J.G.W.); 11 Division of Biostatistics and Epidemiology, Department of Pediatrics, Cincinnati Children's Hospital Medical Center, Cincinnati, OH (J.G.W.); 12 Tulane School of Public Health and Tropical Medicine, New Orleans, LA (W.C., S.R.S., G.B.); 13 Children's Nutrition Research Center, Department of Pediatrics, Baylor College of Medicine, Houston, TX (T.N.); 14 Department of Clinical Physiology and Nuclear Medicine, Turku University Hospital, Finland (O.T.R.)

**Keywords:** cardiovascular, carotid intima‐media thickness, cohort studies, health behavior

## Abstract

**Background:**

Goals for cardiovascular (CV) disease prevention were set by the American Heart Association in 2010 for the concept of CV health. Ideal CV health is defined by 7 CV health metrics: blood pressure, glucose, cholesterol, body mass index, and physical activity on recommended levels; nonsmoking; and a healthy diet. We studied the prevalence of ideal CV health and its associations with ultrasonographically measured carotid intima‐media thickness (cIMT) cross‐sectionally in 5 international populations.

**Methods and Results:**

Prevalence of ideal CV health was assessed among 5785 young adults (age, 36.6±3.2 years) comprising 335 participants from the Minneapolis Childhood Cohort Studies (Minnesota), 723 from the Princeton Follow‐up Study, 981 from the Bogalusa Heart Study (BHS), 1898 from the Cardiovascular Risk in Young Finns Study (YFS), and 1848 from the Childhood Determinants of Adult Health Study (CDAH). Only 1% of the participants had all 7 ideal CV health metrics. The number of ideal CV health metrics associated inversely with cIMT in the 4 cohorts in which cIMT was available: for each additional ideal CV health metric, cIMT was 12.7 μm thinner in Minnesota (*P*=0.0002), 9.1 μm thinner in BHS (*P*=0.05), 10.4 μm thinner in YFS (*P*<0.0001), and 3.4 μm thinner in CDAH (*P*=0.03).

**Conclusions:**

The number of ideal CV health metrics was inversely associated with cIMT in the cohorts in which cIMT was available, indicating that ideal CV health metrics are associated with vascular health at the population level. Ideal CV health was rare in this large international sample of young adults, emphasizing the need for effective strategies for health promotion.

## Introduction

The American Heart Association (AHA) 2020 Strategic Goals included the concept of ideal cardiovascular (CV) health,^1^ with the emphasis placed on the prevention of CV disease by promoting a healthy lifestyle. Ideal CV health is defined as optimal levels for 3 CV risk factors (blood pressure, fasting plasma glucose, and cholesterol) and 4 behaviors (body mass index [BMI], smoking, physical activity, and diet). Recent studies have shown the prevalence of ideal CV health in the United States to be low^2–4^ and associated with subsequent CV disease risk^2^ and mortality.^5^ Because the concept of ideal CV health is relatively new, it has not been widely studied, especially outside of the United States.^6^

The present study was conducted in young adults from 5 international longitudinal populations: (1) the Minneapolis Childhood Cohort Studies (Minnesota, USA), (2) the Princeton Follow‐up Study (Ohio, USA), (3) the Bogalusa Heart Study (Louisiana, USA), (4) the Cardiovascular Risk in Young Finns Study (Finland), and (5) the Childhood Determinants of Adult Health (CDAH) Study (Australia), all members of the International Childhood Cardiovascular Cohort (i3C) Consortium.^7^ Our specific aims were to assess the prevalence of ideal CV health and the relation between ideal CV health and ultrasonographically measured carotid intima‐media thickness (cIMT)^8^ for the combined cohorts and to compare these factors among the studies. Increased carotid IMT is an established marker of subclinical atherosclerosis that is associated with future CV events^9^ and cardiovascular mortality.^10^ Although prior studies have evaluated ideal CV health, its association with cIMT has not been previously reported.

## Methods

### Cohorts and Subjects

The study consisted of 5785 participants from the i3C Consortium.^7^ The cohort studies followed protocols approved by local ethics committees, with signed informed consent. All cohorts had data collected in clinical examinations on the participants' age, sex, race (white or other), height and weight, blood pressure, total cholesterol, and glucose. Self‐reported questionnaires were used to derive data on tobacco smoking, physical activity, and diet. Detailed descriptions of the studies, including attrition analyses demonstrating the representativeness of the cohorts, have been previously published.^11–17^

### Minneapolis Childhood Cohort Studies

The Minneapolis Childhood Cohort Studies (Minnesota) consist of 3 separate cohort studies conducted in Minneapolis and St. Paul, Minnesota, with initial recruitment of schoolchildren (age, 7 to 15 years) in 1978, 1985, and 1995; repeated examinations of anthropometric and clinical measures and vascular studies in the third–fourth decades. The study protocols have been described in detail elsewhere.^18–20^ The present study includes 335 adult participants from Minnesota with data on all ideal CV health metrics and cIMT. Diet was assessed with a 151‐item Youth/Adolescent Questionnaire (YAQ), a self‐administered, semiquantitative food frequency questionnaire (FFQ) developed by the Harvard School of Public Health and Brigham and Women's Hospital.

### Princeton Follow‐up Study

The Princeton Follow‐up Study (PFS) was conducted in 1998–2003 to reexamine lipids and other risk factors of CV disease in an established cohort during participants' fourth decade of life. The participants were originally seen in 1973–1978 as schoolchildren in the Princeton City School District (Cincinnati, OH, USA) as part of the National Heart, Lung, and Blood Institute Lipid Research Clinics (LRC). Detailed protocols of the LRC and PFS have been described previously.^21–22^ The present study includes 723 participants in the PFS, with physical examinations and questionnaire data concerning physical activity, smoking, and other lifestyle factors. Diet was assessed using a 110‐item Block FFQ.^23^ In the PFS, cIMT measurements were not performed.

### Bogalusa Heart Study

The Bogalusa Heart Study (BHS) began in 1973 as an epidemiological investigation of cardiovascular risk factors and their environmental determinants in a black and white pediatric population of the semirural community of Washington Parish, Bogalusa (LA, USA). The study was later expanded to include follow‐up observations of young adults.^24^ Data for the present study were collected during a follow‐up cross‐sectional survey of 981 young adults aged 20 to 38 years conducted in 1995–1996, when carotid ultrasound measurements were performed for 332 participants. Anthropometric and laboratory measures were taken, and the participants responded to a 131–food item YAQ.

### Cardiovascular Risk in Young Finns Study

The Cardiovascular Risk in Young Finns study (YFS) is an ongoing follow‐up study of atherosclerosis risk factors in 5 study centers in Finland (Turku, Helsinki, Tampere, Kuopio, and Oulu). The details of the study design and methods have been published previously.^15^ The first cross‐sectional survey was conducted in 1980, when 3596 children and adolescents aged 3, 6, 9, 12, 15, and 18 years were examined according to a standardized protocol. Follow‐up examinations took place in 1983, 1986, 2001, and 2007. The present analyses are based on the latest follow‐up in 2007, when 1898 persons participated in the clinical examinations and responded to the questionnaires about smoking, physical activity, and general health and 1893 had measurements of cIMT. Diet was assessed using a modified 131‐item FFQ developed by the Finnish National Institute for Health and Welfare.^25^

### Childhood Determinants of Adult Health Study

The Childhood Determinants of Adult Health Study (CDAH) collected information on CV risk factors and levels of fitness, health, and physical performance in examinations of Australian schoolchildren in 1985. The CDAH follow‐up was conducted from 2004 to 2006, when clinic measures were collected and cIMT was measured with ultrasound. The present analyses are based on 1848 participants who had complete data from clinical measurements and smoking and physical activity information from questionnaires. Diet was assessed using a modified 127‐item FFQ and a food habits questionnaire.^26^ Ultrasound measurements of cIMT were performed for 1584 of the participants.

### Assessing the Ideal Cardiovascular Health Metrics

AHA guidelines were used to construct an ideal CV health index of 7 metrics, with the participants given 1 point for the presence of each ideal metric. The ideal health factors defined by AHA were as follows: systolic blood pressure <120 mm Hg and diastolic blood pressure <80 mm Hg; total cholesterol ≤5.17 mmol/L (≤200 mg/dL); fasting glucose <5.6 mmol/L (<100 mg/dL). The ideal health behaviors were classified as BMI <25 kg/m^2^; ≥150 min/week moderate or ≥75 min/week vigorous physical activity or combination; and not smoking (either never having smoked or quit smoking >12 months ago).

Heterogeneity of data collection among the cohorts required some modifications of the AHA definitions (Table 1). Because data on ever‐smokers who quit >12 months ago were missing from Minnesota, we classified only never‐smokers as ideal for this cohort. Time since quitting smoking was only recorded up to 12 months in the BHS, so that only current smokers were classified as nonideal.

**Table 1.** Description of Ideal Cardiovascular Health Metrics

Metric	AHA Criterion	Minnesota	PFS	BHS	YFS	CDAH
Ideal health factors						
Blood pressure, mm Hg	120/80	120/80	120/80	120/80	120/80	120/80
Total cholesterol, mmol/L	≤5.17	≤5.17	≤5.17	≤5.17	≤5.17	≤5.17
Glucose, mmol/L	<5.6	<5.6	<5.6	<5.6	<5.6	<5.6
Ideal health behaviors						
BMI, kg/m^2^	<25	<25	<25	<25	<25	<25
Nonsmoking	Never smoked or quit >1 year ago	Never smoked	Never smoked or quit >1 year ago	Currently not smoking	Never smoked or quit >1 year ago	Never smoked or quit >1 year ago
Physical activity	≥150 min/week moderate or ≥75 min/week vigorous or combination	≥150 min/week combination or ≥75 min/week vigorous	≥150 min/week combination or ≥75 min/week vigorous	≥150 min/week combination	≥120 min/week moderate or combination or ≥60 min/week vigorous	≥150 min/week moderate or combination or ≥75 min/week vigorous
Diet	4 of 5 Components expressed for a 2000‐kcal diet	2 of 3 Components scaled for caloric intake	4 of 5 Components scaled for caloric intake	4 of 5 Components scaled for caloric intake	4 of 5 Components scaled for caloric intake	3 of 4 Components
Fruits and vegetables	≥4.5 Cups per day	Not available	≥4.5 Servings/day	≥450 g/day	≥450 g/day	≥4.5 Servings/day
Fish	≥Two 3.5‐oz servings/week	Saturated fat intake <7 E%	Saturated fat intake <7 E%	Saturated fat intake <7 E%	≥2 Servings (100 g)/week	≥2 Fin fish servings/week
Whole grains	≥Three 1‐oz servings/day	≥3 Servings (30 g)/day	≥3 Servings (30 g)/day	≥3 Servings (30 g)/day of cooked breakfast cereals, dark bread, one third cornbread, one third pasta	≥3 Servings (30 g)/day of whole‐grain rye bread	≥3 Servings/day
Sodium, mg/day	<1500	<1500	<1500	<1500	<1500	Not available
Sugared drinks	≤450 kcal/week	Not available	Sugar from sweets ≤12.8 E%/day	≤450 kcal/week	≤450 kcal/week	≤4 servings/week

AHA indicates American Heart Association; Minnesota, Minneapolis Childhood Cohort Studies; PFS, Princeton Follow‐up Study; BHS, Bogalusa Heart Study; YFS, Young Finns Study; CDAH, Childhood Determinants of Adult Health Study; BMI, body mass index; E%, percentage of total energy intake.

The AHA dietary intake goals were expressed for a 2000‐kcal diet, and we scaled the food and nutrient intakes for energy intake in Minnesota, PFS, BHS, and YFS. Energy intake was not available for the CDAH. The AHA criteria of ideal diet include 4 of the following 5 requirements: ≥450 g of fruits and vegetables consumed per day; ≥two 100‐g servings of fish per week; ≥three 30‐g servings of whole grains per day; ≤450 kcal from sugared drinks per week; and intake of sodium <1500 mg/day (Table 1). To have an ideal diet, at least 4 of 5 diet components (PFS, BHS, YFS), 3 of 4 diet components (CDAH), or 2 of 3 diet components (Minnesota) were required. Fish consumption was not available from the PFS and BHS, and a low saturated fat intake (<7% of total energy intake) was used as a surrogate because saturated fat intake was included as a secondary metric in the AHA's dietary criteria. Consumption of sugared drinks was not available from Minnesota and the PFS. In the PFS, the percentage of total energy intake of sugar from sweets was used instead. Modifications in the consumption of whole grains were done in the BHS to include cooked breakfast cereals, dark bread, and one third each of cornbread and pasta and in the YFS to include whole‐grain rye bread only (contributing the major part of the whole grain consumption) because of limitations in the composition database food grouping.

### Measurement of cIMT

In Minnesota and the YFS the measurement of cIMT was performed with Acuson Sequoia 512 ultrasound scanners (Siemens Medical Solutions USA Inc, Mountain View, CA). In the Minnesota cohort an 8.0‐MHz linear array transducer was used, and cIMT was measured in the common carotid approximately 5 to 11 mm proximal to the carotid bulb.^27^ In the YFS, 13.0‐MHz linear array transducers were used, and mean cIMT was calculated from 4 measurements of the far wall of the left common carotid ≈10 mm proximal to the bifurcation.^28^

In the CDAH, cIMT was measured using a portable Acuson Cypress ultrasound scanner with a 7.0‐MHz linear array transducer following the standardized imaging protocols used in the YFS.^29^ Six measurements of the common carotid far wall were taken approximately 10 mm before the border of the carotid bulb to derive mean cIMT.

In the BHS, cIMT was measured with a Toshiba Ultrasound instrument (Power Vision Toshiba SSH‐380 Digital Ultrasound System, Toshiba America Medical Systems, Carrollton, TX), using a 7.5‐MHz linear array transducer in the far wall of the left common carotid.^30^ In the PFS, cIMT was not measured.

### Statistical Methods

Demographic and clinical characteristics across cohorts were compared with analysis of variance for continuous variables and the χ^2^ test for categorical variables. Associations between the number of ideal CV health factors (blood pressure, glucose, cholesterol) and behaviors (BMI, physical activity, nonsmoking, diet) were studied with Spearman's correlation. The effect of the number of ideal CV health metrics on cIMT was studied with linear regression. To test the independent effects of the ideal CV health metrics on cIMT in multivariable linear regression adjusting for age, sex, race, and cohort, we first tested each metric separately and then in a mutually adjusted model including all 7 ideal CV health metrics. To examine differences in the effect of the number of ideal CV health metrics on cIMT between cohorts, we used the term *ideal CV health*×*cohort* in the pooled analysis and interactions between the individual metrics in cohort‐stratified models. An interaction between the number of ideal CV health factors and behaviors on cIMT was studied with the interaction term *factors×behaviors*. A 2‐sided *P*<0.05 was considered statistically significant. The analyses were performed with SAS version 9.3.

## Results

**Figure 1.**
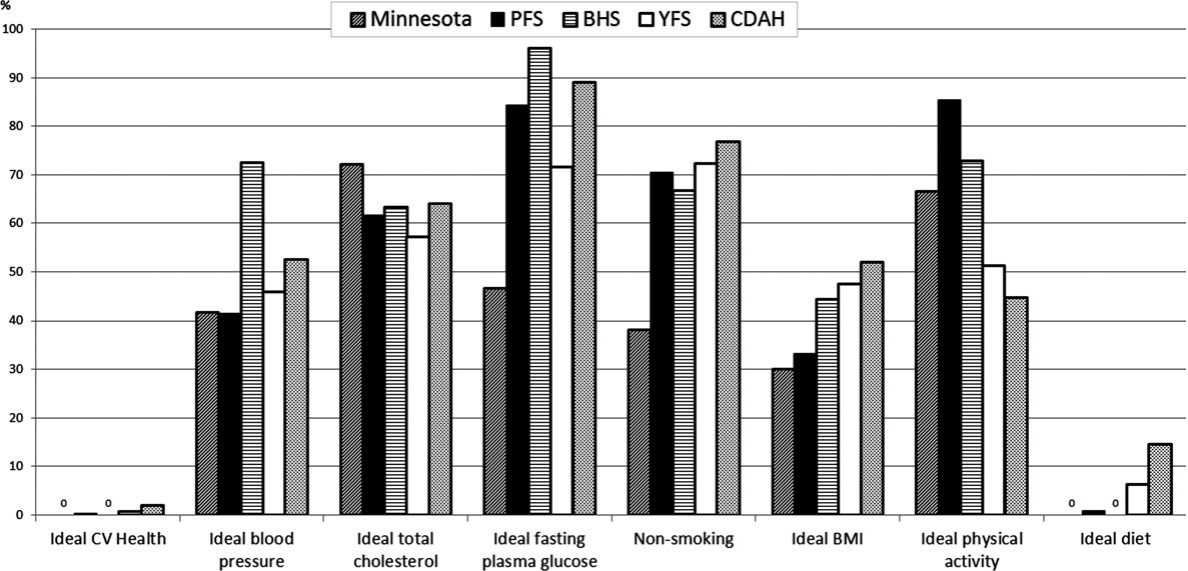
Proportions of participants with the ideal cardiovascular health metrics among the Minnesota, PFS, BHS, YFS, and CDAH cohorts. Zeros indicate 0% prevalence. Minnesota indicates Minneapolis Childhood Cohort Studies; PFS, Princeton Follow‐up Study; BHS, Bogalusa Heart Study; YFS, Young Finns Study; CDAH, Childhood Determinants of Adult Health Study; CV, cardiovascular; BMI, body mass index.

**Figure 2.**
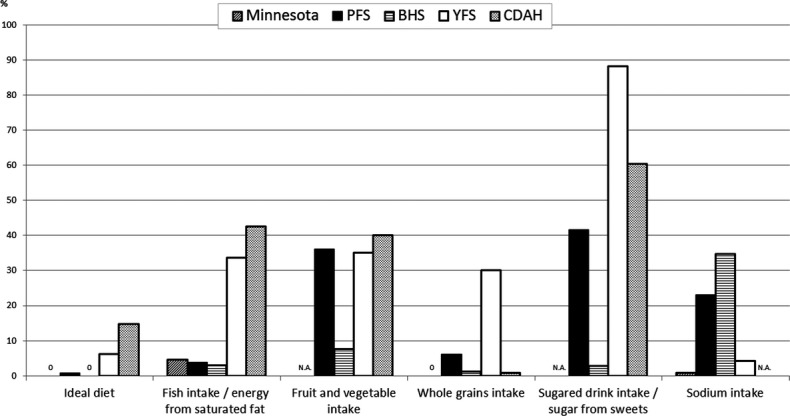
Proportions of participants with the ideal diet components among the Minnesota, PFS, BHS, YFS, and CDAH cohorts. Zeros indicate 0% prevalence, and missing data are denoted by NA (not available). Minnesota indicates Minneapolis Childhood Cohort Studies; PFS, Princeton Follow‐up Study; BHS, Bogalusa Heart Study; YFS, Young Finns Study; CDAH, Childhood Determinants of Adult Health Study.

Characteristics of the participants are displayed in Table 2. Only 1.0% of the participants from the combined cohorts had all 7 ideal health metrics (range across cohorts, 0% to 2.0%; Figure 1). Ideal fasting plasma glucose (82%; range across cohorts, 47% to 96%), nonsmoking (70%; range across cohorts, 38% to 77%), ideal total cholesterol (62%; range across cohorts, 57% to 72%), and ideal physical activity (58%; range across cohorts, 46% to 85%) were found most commonly, and ideal diet (7%; range across cohorts, 0% to 15%), ideal BMI (46%; range across cohorts, 30% to 52%) and ideal blood pressure (52%; range across cohorts, 41% to 72%) least commonly among the participants. The proportions of individuals achieving the individual diet components among the cohorts are presented in Figure 2. Ideal diet was rare, and therefore we also calculated the percentage of participants who had 6 ideal health components, omitting the ideal diet. For pooled data, 9% had this modified ideal health, and for the individual cohorts prevalence was 4% in Minnesota, 11% in PFS, 13% in BHS, 7% in YFS, and 9% in CDAH.

**Table 2.** Descriptive Characteristics (Mean±SD or %) of the Study Populations

Variable	Minnesota	PFS	BHS	YFS	CDAH	All
Participants in adulthood, n	335	723	981	1893	1848	5785
Age, y	39.2±1.5	38.5±3.6	29.5±5.1	37.8±5.0	31.0±2.7	34.4±5.6
Men, %	50	45	37	44	48	45
White, %	65	71	77	100	100	90
BMI, kg/m^2^	29.3±7.4	28.7±6.9	27.2±6.8	25.9±4.7	25.6±4.8	26.6±5.8
Systolic blood pressure, mm Hg	125±16	121±15	110±11	120±14	118±13	118±14
Diastolic blood pressure, mm Hg	72±10	80±11	73±9	75±11	72±9	74±10
Total cholesterol,* mmol/L	4.8±0.8	5.0±1.0	4.9±1.0	5.0±0.9	4.9±1.0	5.0±1.0
Glucose,* mmol/L	5.9±1.9	5.0±1.5	4.5±0.6	5.3±0.9	5.0±0.5	5.1±1.0
Daily smoking, %		31	36	28	23	30
Ever smoking, %	64*	—	—	—	—	—
Carotid IMT, μm	519.7±83.0	NA	667.3±103.0*	626.4±96.0*	561.5±85.2*	596.3±100.7*

Differences across cohorts, *P*<0.0001 (except for percentage of men, *P*=0.04). Minnesota indicates Minneapolis Childhood Cohort Studies; PFS, Princeton Follow‐up Study; BHS, Bogalusa Heart Study; YFS, Young Finns Study; CDAH, Childhood Determinants of Adult Health Study; BMI, body mass index; IMT, intima‐media thickness; SD, standard deviation; NA, not available.

* Divide by 0.0259 to transform to mg/dL.

* Divide by 0.0555 to transform to mg/dL.

* Daily smoking was not available for Minnesota.

* n=332.

* n=1893.

* n=1584.

* n=4144.

The number of ideal CV health behaviors (BMI, physical activity, smoking, and diet) was directly significantly correlated with the number of ideal health factors (blood pressure, glucose, and cholesterol) in each of the cohorts (Spearman's *r*=0.20, *P*=0.0002 in Minnesota; *r*=0.26, *P*<0.0001 in PFS; *r*=0.15, *P*<0.0001 in BHS; *r*=0.23, *P*<0.0001 in YFS; and *r*=0.21, *P*<0.0001 in CDAH).

**Figure 3.**
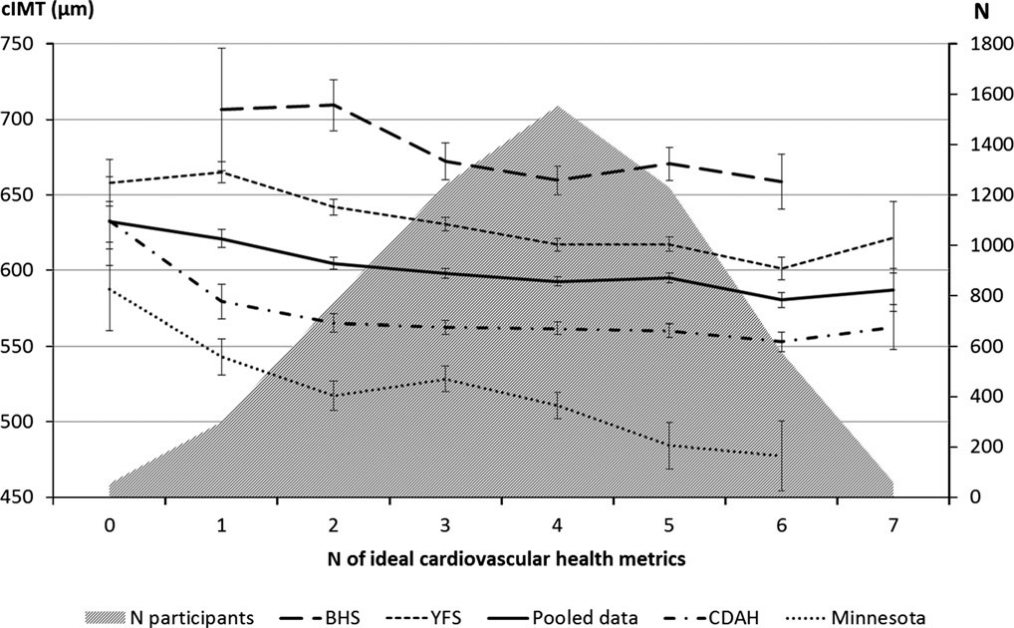
Age‐ and sex‐adjusted mean±SE carotid intima‐media thickness (cIMT, μm) according to the number of ideal cardiovascular health metrics in pooled data (n=4144) and in the Minnesota (n=335), BHS (n=322), YFS (n=1893), and CDAH (n=1584) cohorts. Minnesota indicates Minneapolis Childhood Cohort Studies; BHS, Bogalusa Heart Study; YFS, Young Finns Study; CDAH, Childhood Determinants of Adult Health Study.

Figure 3 shows mean cIMT by number of ideal CV health metrics for the combined cohorts and for each of the individual cohorts. There was a similar pattern of differences in cIMT over the number of ideal CV health metrics, and the main difference was in the level of cIMT across cohorts. The interaction between the number of ideal CV health and cohort (*P*=0.001) was likely significant because of the large sample size. For each additional ideal CV health metric, cIMT was 12.7 μm thinner in Minnesota (*P*=0.0002), 9.1 μm thinner in BHS (*P*=0.05), 10.4 μm thinner in YFS (*P*<0.0001), and 3.4 μm (*P*=0.03) thinner in CDAH. In the pooled data, cIMT was 6.6 μm thinner (*P*<0.0001) for each additional ideal CV health metric.

Multivariable effects of the ideal CV health metrics on cIMT in age‐, sex‐, race‐, and cohort‐adjusted multivariable models are shown in Table 3. The associations were first studied separately for each of the ideal CV health metrics and then in mutually adjusted models including all metrics. Ideal blood pressure, ideal cholesterol, ideal BMI, and ideal diet were independently inversely associated with cIMT in the separate analyses. In the mutually adjusted analysis, blood pressure, cholesterol, and BMI were inversely associated with cIMT, whereas physical activity was directly associated with cIMT. Ideal glucose (*P*=0.05), nonsmoking (*P*=0.08), and ideal diet (*P*=0.05) were approaching significance. The ideal physical activity and ideal glucose metrics were directly associated with nonsmoking and ideal diet inversely associated with cIMT in both models. The multivariable analyses stratified by cohort are presented in Table 4.

**Table 3.** Independent Effects of Ideal Cardiovascular Health Metrics on cIMT (in micrograms) for Each Metric in Pooled Data (n=4141)

	Model I		Model II	
	β±SE	*P* Value	β±SE	*P* Value
Ideal blood pressure	−16.1±3.2	<0.0001	−12.1±3.2	0.0002
Ideal total cholesterol	−15.2±3.1	<0.0001	−11.8±3.1	0.0001
Ideal glucose	2.5±3.9	0.52	7.7±3.9	0.05
Nonsmoking	−5.3±3.3	0.11	−5.8±3.3	0.08
Ideal BMI	−22.6±3.0	<0.0001	−20.0±3.1	<0.0001
Ideal diet	−11.2±5.4	0.04	−10.5±5.4	0.05
Ideal physical activity	7.7±3.0	0.008	9.3±3.0	0.002

Model I adjusted for age, sex, cohort, and race, and Model II mutually adjusted for all variables and for age, sex, cohort, and race (*R*^2^=0.126). cIMT indicates carotid intima‐media thickness; BMI, body mass index.

**Table 4.** Multivariable Correlates of cIMT in the Minnesota, BHS, YFS, and CDAH Cohorts

Covariate	Minnesota (n=335)		BHS (n=332)		YFS (n=1893)		CDAH (n=1584)	
	β±SE	*P* Value	β±SE	*P* Value	β±SE	*P* Value	β±SE	*P* Value
Age	8.7±2.9	0.003	4.7±1.8	0.007	5.8±0.4	<0.0001	4.9±0.8	<0.0001
Female sex	−13.1±9.1	0.15	−28.7±11.7	0.01	−15.0±4.4	0.0007	−23.3±4.8	<0.0001
Ideal blood pressure	−27.0±9.3	0.004	−12.7±12.5	0.31	−24.4±4.4	<0.0001	−5.8±4.8	0.23
Ideal total cholesterol	5.3±10.0	0.60	−8.0±11.5	0.49	−4.0±4.3	0.35	−5.8±4.4	0.19
Ideal glucose	−3.7±9.1	0.69	−30.6±31.7	0.34	−2.0±4.7	0.67	−4.8±6.9	0.49
Nonsmoking	−17.3±8.9	0.05	−12.5±11.7	0.29	−4.8±4.6	0.29	−10.3±5.0	0.04
Ideal BMI	−27.2±10.1	0.007	−16.5±12.1	0.18	−25.6±4.3	<0.0001	−7.4±4.4	0.09
Ideal diet	—	—*	54.4±101.4	0.59	−7.2±8.4	0.39	8.1±6.0	0.18
Ideal physical activity	4.4±9.1	0.63	6.2±12.5	0.62	0.7±4.1	0.86	4.5±4.2	0.29

β refers to the effect on cIMT (μm) for each additional ideal cardiovascular health metric. cIMT indicates carotid intima‐media thickness; Minnesota, Minneapolis Childhood Cohort Studies; PFS, Princeton Follow‐up Study; BHS, Bogalusa Heart Study; YFS, Young Finns Study; CDAH, Childhood Determinants of Adult Health Study; BMI, body mass index.

* Ideal diet metric=0 for all Minnesota participants.

**Figure 4.**
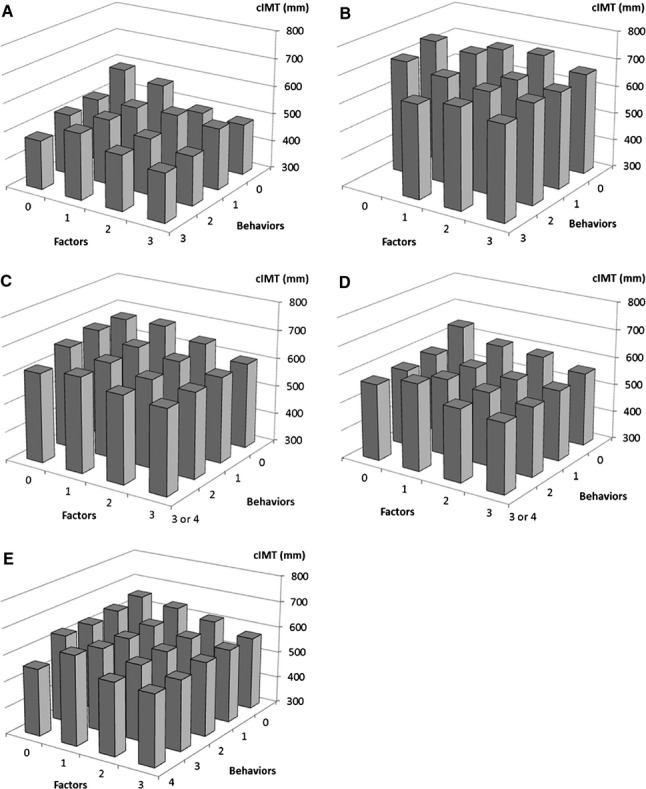
Carotid intima‐media thickness (cIMT, μm) per number of ideal health behaviors and factors in (A) Minnesota; (B) BHS; (C) YFS; (D) CDAH; and (E) pooled cohorts. Minnesota indicates Minneapolis Childhood Cohort Studies; BHS, Bogalusa Heart Study; YFS, Young Finns Study; CDAH, Childhood Determinants of Adult Health Study.

Figure 4 shows mean cIMT by the number of ideal health behaviors and factors among the Minnesota (A), BHS (B), YFS (C), and CDAH (D) cohorts and in the pooled data (E). Both the number of ideal behaviors and factors were significantly associated with cIMT in the pooled data (Spearman's correlation: factors *r*=−0.17, *P*<0.0001; behaviors *r*=−0.08, *P*<0.0001) and in the Minnesota (factors *r*=−0.19, *P*=0.0004; behaviors *r*=−0.16, *P*=0.003), BHS (factors *r*=−0.17, *P*=0.002; behaviors *r*=−0.11, *P*=0.04), and YFS (factors *r*=−0.24, *P*<0.0001; behaviors *r*=−0.14, *P*<0.0001), but only the health factors showed a significant association with cIMT in CDAH (factors *r*=−0.12, *P*<0.0001; behaviors *r*=−0.05, *P*=0.05). The effect of an interaction between factors and behaviors on cIMT was not significant in pooled data (*P*=0.63), Minnesota (*P*=0.54), BHS (*P*=0.21), YFS (*P*=0.38), and CDAH (*P*=0.66).

## Discussion

The present study shows that ideal CV health, as defined by the American Heart Association, is uncommon among young adult populations in the United States, Finland, and Australia (observed in only 1% of the 5785 young adults participating in the international cohorts). Many of the participants had ideal glucose (73%) and ideal cholesterol (64%) and were currently not smoking (64%). Ideal diet (7%) was the least common metric for participants from any of the cohorts.

This study expands on results from previous studies on ideal CV health by showing an inverse relation to cIMT, a subclinical measure of atherosclerosis, indicating the potential adverse early consequences of elevated CV risk factors in young adulthood. It previously has been shown that low levels of cardiometabolic risk markers in childhood are associated with thinner cIMT and lower prevalence of the metabolic syndrome in adulthood.^31^ The cross‐sectional association between ideal CV health and cIMT in adulthood has not been shown before, and our cohorts were younger in age compared with other available data on the ideal CV health metrics.

The goal of the ideal CV health concept has been to emphasize prevention of CV disease by promoting healthy behaviors.^1^ In this study, very few of the young adults fit within the limits of all 7 health behaviors and factors. This is consistent with recent studies reporting a very low prevalence of ideal CV health using the same metrics. In a cross‐sectional study evaluating the community prevalence of these metrics in the 1933 participants of the Heart Strategies Concentrating on Risk Evaluation study, only 0.1% of the participants met all the ideal metrics of ideal CV health as defined by the AHA.^3^ Of the 9962 Chinese participants in the Disease Risk Evaluation and Health Management study, 0.5% met all 7 ideal CV health metrics.^6^ A prevalence of 0.1% for all ideal metrics was found in the 12 744 participants of the Atherosclerosis Risk in Communities Study.^32^ In the National Health Examination Survey follow‐up of 7622 adults, 1.1% of the subjects had all 7 ideal health metrics.^5^ The 20‐year follow‐up of the 3154 participants in the CARDIA study found that only 6% of the participants had all 5 of the studied healthy behaviors included in that study, and a healthy lifestyle was associated with lower CV disease risk later in life.^2^ The very low prevalence of ideal CV health in the present study and several prior studies suggests that the behavior changes required for compliance with the ideal health behaviors are difficult targets to achieve. When we applied the AHA definitions for the concept of ideal CV health ^1^ for each of the 3 factors and 4 behaviors that separate ideal from nonideal CV health to our young adult cohorts, few met the diet criteria, and therefore only 1% had complete ideal CV health.

Ideal CV health has been associated with CV morbidity in the Northern Manhattan Study, in which the index predicted a lower risk of CVD end points.^33^ Similarly, the number of ideal CV health metrics was associated with lower total and CVD mortality among the 44 959 US adults in the National Health and Nutrition Examination Survey ^34^ and in a second study in the United States.^5^ The YFS study has previously shown that ideal CV health in children predicts CV health outcomes (eg, lower cIMT and a lower risk of hypertension, dyslipidemia, and metabolic syndrome) in adulthood after a 21‐year follow‐up.^35^ The current analysis shows the significant cross‐sectional relation between ideal CV health and cIMT in young adults from cohorts in the United States, Finland, and Australia. Among the examined cohorts, both the total number of ideal CV health metrics and the individual metrics—ideal BMI, ideal blood pressure, ideal total cholesterol, and ideal physical activity—were shown to have independent associations with cIMT.

The low prevalence of ideal CV health may reflect an imbalance in the thresholds for each ideal health metric. Compliance with 2 to 4 of the dietary criteria was required for an ideal diet, which was a more stringent requirement than compliance with the other components having 1 threshold (or 2 in the case of blood pressure). Compliance was particularly rare for low sodium intake and high fish consumption/low saturated fat intake in our cohorts, making the full score of all 7 metrics almost nonexistent. Low saturated fat intake was used as a surrogate when fish consumption was not available, because saturated fat intake was included as a secondary metric in the AHA's dietary criteria.^1^ Although widely applied in population studies, the FFQ method is not ideally suitable for precise measurement of nutrient intakes.^36^ Sodium intake especially may be incorrectly estimated because of the assumptions made in the composition of the food and nutrient database. Therefore, it is possible that some of the subjects actually consumed diets with lower sodium content than estimated here, leading to an underestimation of diet compliance.

The arbitrary cutoffs for the health behaviors and, to a lesser degree, the health factors have also been critically discussed.^1^ Various adjustments to the cutoffs such as 2300 mg sodium, although not ideal, could be regarded as a step in the right direction. One approach to compose a modified healthy dietary score has been to use a Healthy Eating Index,^5^ based on the AHA definitions, including fruits and vegetables, whole grains, and sodium but not including sugar‐sweetened beverages and fish consumption. Others have based their healthy dietary factors on intakes of calcium, potassium, fiber, and saturated fat.^2^ We also calculated the percentage those who had a modified ideal health consisting of 6 metrics (without taking into account the ideal diet metric, and 9% of the participants had this modified ideal health.

Limitations of this study include the cross‐sectional design that prevented analyses related to identification of causal associations, the need to modify some of the AHA's definitions for ideal CV health, the need to use partly different definitions for different cohorts, and the absence of data on some of the components in some cohorts. However, these are offset by the strengths of being able to construct most of the ideal CV health indices according to the AHA definitions and of being able to use cIMT as a marker of vascular health. Differences between the cohorts in measurement methods and time points and in age and racial distributions may explain part of the differences in having the ideal CV health metrics. Another limitation is that cIMT was measured using ultrasonographic transducers of various manufacturers and with different frequencies, ranging between 7 and 13 MHz in the Minnesota, BHS, YFS, and CDAH cohorts, and differences in image quality may explain part of the differences in cIMT levels across cohorts.^37^ However, the direction of effect on cIMT of the number of ideal CV health metrics was the same in all cohorts in which cIMT was available. Therefore, it seems that although there are differences in the ideal CV health metrics and the mean cIMT in different populations, adherence to the components of ideal CV health in young adulthood is universally associated with a lower risk of subclinical atherosclerosis.

## Clinical Implications

The number of ideal CV health metrics was inversely associated with cIMT, suggesting that ideal CV health reflects vascular health at the population level. This finding and the fact that complete ideal CV health was very rare among this large sample of young adults strengthen the need for early evaluation of CV risk factors and for development of effective intervention strategies for behavioral change.
